# Experimental demonstration of a transparent graphene millimetre wave absorber with 28% fractional bandwidth at 140 GHz

**DOI:** 10.1038/srep04130

**Published:** 2014-02-19

**Authors:** Bian Wu, Hatice M. Tuncer, Majid Naeem, Bin Yang, Matthew T. Cole, William I. Milne, Yang Hao

**Affiliations:** 1 School of Electronic Engineering and Computer Science, Queen Mary University of London, London, E1 4NS, United Kingdom; 2 Department of Engineering, University of Cambridge, 9 JJ Thomson Avenue, Cambridge, CB3 0FA, United Kingdom; 3 School of Electronic Engineering, Xidian University, Xi'an, 710071, China; 4 Engineering, Sports & Sciences Academic Group, University of Bolton, Deane Road, Bolton, BL3 5AB, United Kingdom

## Abstract

The development of transparent radio-frequency electronics has been limited, until recently, by the lack of suitable materials. Naturally thin and transparent graphene may lead to disruptive innovations in such applications. Here, we realize optically transparent broadband absorbers operating in the millimetre wave regime achieved by stacking graphene bearing quartz substrates on a ground plate. Broadband absorption is a result of mutually coupled Fabry-Perot resonators represented by each graphene-quartz substrate. An analytical model has been developed to predict the absorption performance and the angular dependence of the absorber. Using a repeated transfer-and-etch process, multilayer graphene was processed to control its surface resistivity. Millimetre wave reflectometer measurements of the stacked graphene-quartz absorbers demonstrated excellent broadband absorption of 90% with a 28% fractional bandwidth from 125–165 GHz. Our data suggests that the absorbers' operation can also be extended to microwave and low-terahertz bands with negligible loss in performance.

Graphene has attracted much attention in recent years due to its extraordinary electronic and optical properties such as high electron mobility, truly atomic thickness, mechanical flexibility and saturable absorption^1^,^2^,^3^,^4^,^5^. With an optical transparency of 97-98%^6^ and an undoped sheet resistance of the order of 6000 Ω/sq^7^ to ~125 Ω/sq^8^, monolayer graphene films have found, not unsurpisingly, many applications where low sheet resistance and high optical transparency are essential. Chemical vapour deposition (CVD) permits the synthesis of high quality, large-area graphene films^9^,^10^,^11^,^12^,^13^ and an increased number of layers provides lower sheet resistances necessary for transparent conducting applications. Few layer graphene films with sheet resistance of 280 Ω/sq (80% transparent) and 770 Ω/sq (90% transparent), synthesized on Ni films^11^,^12^, and 350 Ω/sq (90% transparent) on Cu films^13^, make CVD graphene one of the few viable substitute materials for the mechanically inflexible transparent conducting oxides; indium tin oxide (ITO) or fluorine-doped tin oxide (FTO)^14^,^15^, in almost all transparent electronic devices^16^,^17^. Chemical doping has been shown to further reduce the sheet resistance of graphene films^18^. Until present most advances in graphene devices have focussed on the terahertz and optical frequencies, while in the radio-frequency regime, where the sheet resistivity dominates, the practical applications of graphene has been towards graphene-based FET mixers^19^,^20^,^21^, RF transistors^22^,^23^,^24^ and controllable resistive devices such as metasurfaces^25^,^26^,^27^,^28^,^29^ or absorbers^30^,^31^. However, the experimental demonstration of transparent radio-frequency absorbers, with broadband properties, still requires further research.

Radar absorbing materials (RAMs) significantly reduce an object's observable radar cross-section within specific bandwidths^32^. A Salisbury screen absorber consists of a thin resistive sheet and a ground separated by a quarter wavelength dielectric filler^33^,^34^. It operates by matching the absorber characteristic impedance to free space and the effective open circuit created leads to reduced reflections at the surface. However, the operation is narrowband and suffers from reduced absorption capability if the angle of incidence is not perpendicular to the absorber^32^. Nevertheless, if the number of screens can be increased, using multiple layers of thin resistive sheets with the filling dielectric, one can form a Jaumann absorber, which offers an extended bandwidth and reduced angular sensitivity^32^,^35^. Though very functional, the added layers and consequent increased thickness, make Jaumann screens a bulky alternative. Thus, there is a clear need to reduce the thickness of the broadband absorber components^33^, with materials which have a characteristic impedance of the same order of magnitude as the free space impedance; graphene is one such leading candidate.

In this letter, we report for the first time on the fabrication and characterization of transparent broadband absorbers consisting of several stacked multilayer graphene sheets on quartz substrates backed with a ground plate. The large-area CVD multilayer graphene films have been fabricated through a repeated etch-and-transfer process to minimise PMMA residue between the layers, thereby yielding high-quality and optically transparent multilayer graphene films. Herein, analytical expressions are developed for the reflection and absorption coefficients consisting of an arbitrary number of stacked graphene layers. These expressions serve as a practical design guide to estimate the influence of the sheet resistance and substrate parameters, as well as the impact of oblique incidence. To validate the broadband absorption, millimetre wave reflectometer experiments have been carried out with single and stacked absorbers from 110 GHz to 170 GHz. For 5-unit stacked structures, 90% absorbance can be achieved with 28% fractional bandwidth from 125 GHz to 165 GHz. The broadband absorption can be further improved by using thinner substrates with lower relative permittivity or stacking more graphene-quartz substrates at the expense of reduced optical transparency.

## Results

### Fabrication and modelling of the stacked graphene-quartz absorber

**Figure 1:**
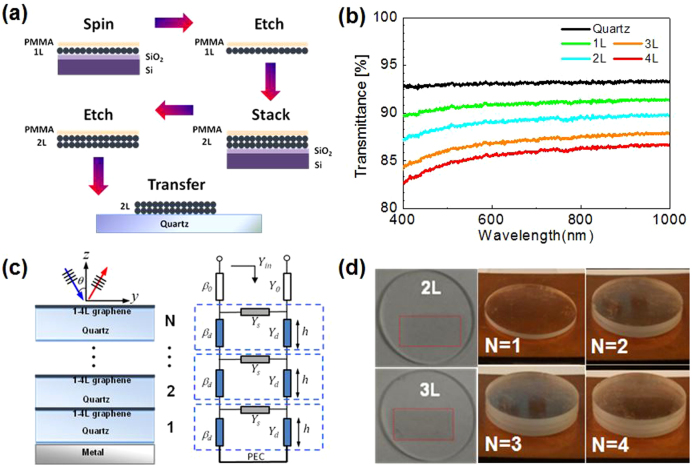
Schematic and optical images of multilayer graphene on quartz and stacked graphene-quartz absorbers. (a) Schematic of the multiple transfer-etch processing for a 2 L device; (b) Typical UV-Vis spectra for the 1.3 mm thick bare quartz and 1–4 L graphene samples; (c) Schematic of the N-unit stacked absorber and the equivalent transmission-line circuit model; (d) Optical images of 2 L and 3 L absorbers and N = 1–4 stacked graphene-quartz structures backed with a ground plate (N is the number of stacked graphene-quartz units).

CVD graphene films were grown on four inch Cu/SiO_2_/Si wafers and were found, by optical and electron microscopy to be free of pin-holes. Samples were of high uniformity with >90% monolayer coverage, as confirmed by Raman spectroscopic mapping and optical microscopy^36^. Films were transferred to fused silica quartz substrates using spin-coated 200 nm thick poly (methyl methacrylate) (PMMA) as the supporting layer (for details see Methods section) (Fig. 1a). Multilayer graphene samples were processed by a multiple transfer-and-etch method. This involves repetitive transfer of the PMMA-graphene films onto diced graphene on Cu/SiO_2_/Si substrates and etching them in an aqueous ammonium persulfate solution before finally transferring the released PMMA/graphene onto the quartz substrates. This method avoids significant PMMA residue build-up between the stacks of graphene layers yielding reduced mean sheet resistance of ~0.9 kΩ/sq for 2 L and ~0.6 kΩ/sq for 3 L. The number of graphene layers was confirmed via UV-Vis spectro-photometery. Optical transmittances of 85%–91% at 700 nm for quartz-supported 1–4 L graphene was noted (Fig. 1b).

The proposed broadband absorber was realized by stacking multilayer graphene bearing quartz samples on top of a ground plate, as depicted in Fig. 1c. Fig. 1d illustrates optical images of quartz-supported 2 L and 3 L graphene layers (17 mm × 8.5 mm). The N-unit samples, with similar sheet resistances, were stacked onto a ground plate to construct the broadband absorbers, as shown in Fig. 1d.

To predict the absorber performance, we have derived an analytical expression based on a circuit model equivalent, as shown in Fig. 1c. For simplicity, it is assumed that all of the graphene sheets have the same surface conductivity *σ_s_* and the quartz substrates are homogeneous and isotropic with a permittivity of *ε_r_* and a thickness of *h*. The absorber model is assumed infinite in the xy plane and stacked along the z direction. A uniform transverse electromagnetic (TEM) plane wave is assumed to arrive at the absorber surface with an oblique incidence angle of *θ*. Since the thickness of quartz is a few millimetres, high-order modes become evanescent and can be ignored in the millimetre wave regime. The graphene sheets can be modelled as thin, two-sided surfaces characterized by surface conductivity, which is governed by the intraband contributions at low-terahertz frequencies and can be expressed as^37^

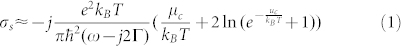
where *ω* is the radian frequency, *μ_c_* is the chemical potential, Γ is the phenomenological scattering rate, *T* is temperature, *e* is the charge of an electron, 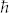 is the reduced Planck's constant and *k_B_* is the Boltzmann's constant. Although *σ_s_* is frequency dependent in equation (1), in the millimetre wave region the conductance term remains almost constant, whilst the susceptance term tends to 0 and can thus be neglected; *σ_s_* is dominated by the surface conductance and can be regarded as frequency independent.

In terms of TEM transmission line theory, the quartz substrate can be modelled as a dielectric with propagation constant *β_d_* and characteristic admittance *Y_d_*, with the resistive graphene sheets represented as a shunt admittance, *Y_s_* = *σ_s_* ≈ 1/*R_s_*, where *R_s_* is the sheet resistance (Fig. 1c). The propagation constant of free space is denoted by *β*_0_ and the characteristic admittance by *Y*_0_. The general analytical expressions for TE and TM polarizations of a wave with incident angle *θ* can be expressed as^38^

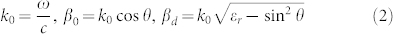

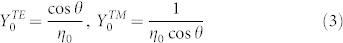

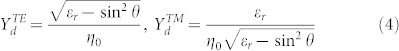
where *c* is the speed of light in vacuum, *ω* is the angular frequency, and 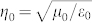 is the intrinsic free space wave impedance.

For a single (N = 1) graphene-quartz absorber, the input admittance is given by 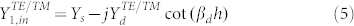
While for the N-unit stacked graphene-quartz absorber, the input admittance can be derived as 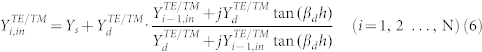
Since there is no transmission due to total reflection on the ground surface, we obtain the reflection coefficient *S*_11_ and absorption coefficient *A* of the stacked graphene-quartz absorber from 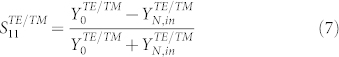

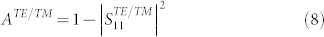


The highest absorption is achieved when *Y_N_*_,*in*_ ≈ *Y*_0_ or *S*_11_ ≈ 0; the absorption peaks correspond to reflection zeros. In this case the incident wave will go through multiple reflections and be fully absorbed by the lossy resistive graphene sheets.

### Measurement and prediction of transparent graphene-quartz absorbers

**Figure 2:**
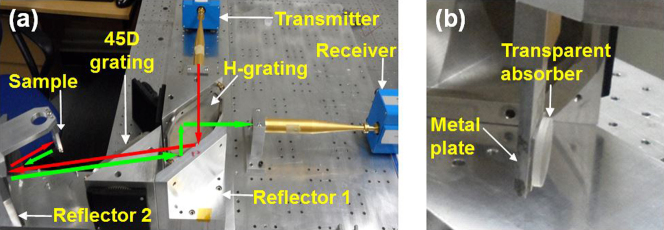
Millimetre wave reflectometer measurements. (a) Photograph of the experimental set-up. Red lines refer to the incident wave from the transmitter to the sample; green lines represent the reflected wave from the sample to the receiver. The H-grating transmits vertically polarized waves but reflects horizontally polarized waves. The 45D grating selects the E-field components with 45° rotation. (b) Photograph of the transparent absorber consisting of graphene-quartz samples backed with a metal plate.

In order to investigate the nanostructured absorber, reflection spectra are measured by a well-established free space millimetre wave reflectometery technique and then transformed to absorption spectra according to equation (8). The experimental set-up is illustrated in Fig. 2a. The reflectometer functions at frequencies from 110 GHz to 170 GHz using a HP N5244A vector network analyzer fitted with millimetre wave extension heads (see Methods section).

Firstly, single (N = 1) absorbers with 1–4 L graphene were measured and then two graphene-quartz samples were stacked together to construct a 2-unit (N = 2) absorber (Fig. 2b). Measurements up to N = 5 were performed. The stacked graphene-quartz structures were backed with a conducting ground plate which was fixed to the metal support, guaranteeing that the sample was perpendicular to the incident wave. Only the normal incidence is considered here. Each graphene adlayer was 17 mm × 8.5 mm and covered the beam width of the incidence wave.

**Figure 3:**
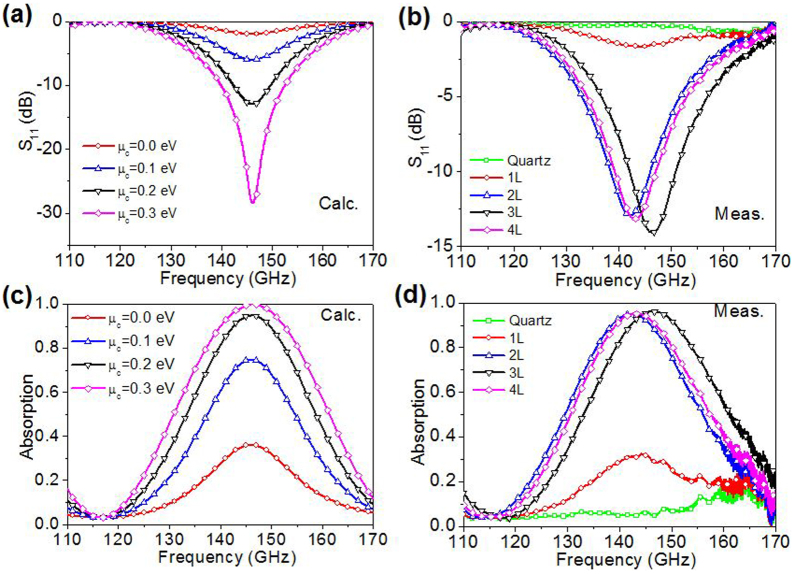
Comparison of calculated and measured spectra of single graphene-quartz absorbers. (a, c) Calculated absorption spectra. The scattering rates are chosen as Γ = 7 meV for *μ_c_* = 0.0 eV and Γ = 5 meV for all others, *T* = 300 K. (b, d) Measured reflection and absorption spectra of single (N = 1) graphene-quartz absorbers with 1–4 L graphene on quartz (*ε_r_* = 3.8 and *h* = 1.3 mm). The measurements show that the improvement in absorption is substantial from 1 L to 2 L multilayer graphene while less significant after 2 L. The measured reflection and absorption spectra in (b) and (d) are similar to calculated spectra in (a) and (c).

The measured reflection and absorption spectra of single graphene-quartz absorbers with 1–4 L graphene on quartz are compared with analytical calculations in Fig. 3. The calculated results in Fig. 3a and 3c show the influence of the chemical potential on the reflection and absorption properties of the absorber. When *μ_c_* = 0 eV and Γ = 7 meV, this corresponds to a sheet resistance of 5044 Ω/sq which makes the input impedance challenging to match with free space. The peak absorption is lower than 40% indicating a poor absorption. As the chemical potential increases, in steps of *μ_c_* = 0.1 eV, the sheet resistance of graphene is reduced and the peak absorption improves. When *μ_c_* = 0.3 eV and Γ = 5 meV, the corresponding sheet resistance is 430 Ω/sq, which tends toward the free space impedance. Good impedance matching leads to 100% peak absorption around 148 GHz.

Graphene surface conductivity is mainly dominated by the intraband contribution from microwaves to far infrared waves. The real part of the intraband conductivity, which contributes to energy absorption or dissipation^39^, increases linearly with the chemical potential at 10 GHz^37^. Our calculations show that this is also the case for the frequency of operation at 140 GHz. A linear increase in surface conductivity can also be achieved by artificially stacking graphene monolayers^40^. We note that the measured absorption improvement of multilayer graphene in Fig. 3d is similar to the calculated absorption curves when the chemical potential is varied from 0–300 meV in Fig. 3c. The chemical potential can be changed either by chemical doping or by the application of a bias voltage^37^,^18^,^40^. Here, we achieve a similar increase in the absorption capability simply by increasing the number of the stacked layers.

First, the bare quartz substrate on the ground plate was tested in the reflectometer as a background reference. A total reflection with no absorption is observed across the whole frequency range, except for the intrinsic systematic noise at high frequencies (>160 GHz). The single unit graphene-quartz absorbers with 1–4 L graphene were then measured. A 1 L absorber has a small absorption peak around 30% at 148 GHz, which is similar to the case of *μ_c_* = 0.0 eV and Γ = 7 meV (Fig. 3c, d). In contrast, a 2 L absorber has a peak absorption around 95%, which is similar to the calculated case of *μ_c_* = 0.2 eV and Γ = 5 meV. A small frequency shift is caused by the thickness variation in the practical quartz slabs (±2%) and the air gap (~0.1 mm) between quartz and ground plate. The absorption peak increase marginally (+1.2%) for the 3 L case and fall slightly (−1%) for the 4 L case. Multilayer graphene can thusly be used to derive a turbostratic, stacked, artificial graphite-like material of sufficiently reduced sheet resistance capable of near matching the free space impedance; however, it is challenging to improve the sheet resistance further for samples consisting of more than 3 layers, which could be due to water residue between the layers that prevents good contact between the interfacing layers in the present samples.

The single graphene-quartz absorber is based on a Salisbury screen absorber (see Supplementary Fig. S1), which exhibits fundamental Fabry-Perot resonance at 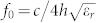, and the incident waves and reflected waves at *f_i_* = (2*i* − 1)*f*_0_ (where *i* = 1, 2 …) have opposite phase difference which leads to periodic reflection zeros and absorption peaks^38^.

Although a Salisbury screen absorber has multiple absorption bands, maximum absorption is limited to a narrow bandwidth around the resonant frequency. In order to achieve wideband absorption we instead consider a Jaumann absorber, which utilizes a series of a quarter wavelength thick dielectric layers that separate parallel resistive sheets, introducing a mutual coupling of Fabry-Perot resonators over a metallic ground. Here we use stacked graphene-quartz samples to realize a transparent millimetre wave Jaumann absorber. The zero-degree reflection phase condition can be used to predict the locations of absorption peaks and zeros (Supplementary Fig. S2). For the N-unit stacked absorber, the location of absorption peaks and zeros in the first band can be calculated from *f_p_*_,*i*_ = (2*i* − 1)*f*_0_/*N* and *f_z,i_* = 2*if*_0_/*N* (*i* = 1, 2 …), respectively. As shown in Fig. S3, the chemical potential of graphene has a remarkable influence on the absorption spectra of the stacked absorber. Instead of tuning the chemical potential, multilayer graphene films were utilized to achieve the required sheet resistance of <1000 Ω/sq.

**Figure 4:**
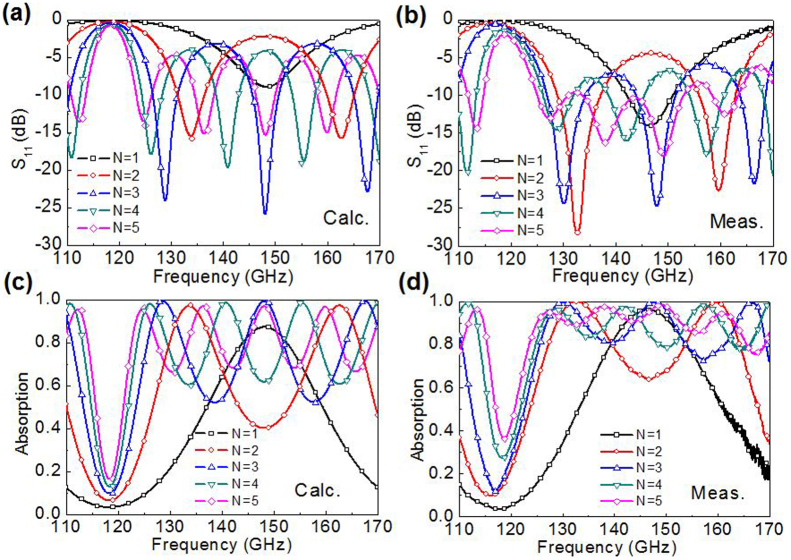
Comparison of calculated and measured spectra of stacked graphene-quartz absorbers. (a, c) Calculated reflection and absorption spectra showing a centre frequency at 148 GHz and an increased number of reflection zeros (or absorption peaks) as well as absorption bandwidth from N = 1 to N = 5, where N is the number of stacked graphene-quartz units. All graphene sheets are assumed to have the same parameters (*μ_c_* = 0.15 eV, Γ = 5 meV) and separated by homogeneous quartz substrate (*ε_r_* = 3.8 and *h* = 1.3 mm). (b, d) Measured reflection and absorption spectra show similar responses as the calculations. The observed slight frequency shift and amplitude variation are mainly associated with parameter differences between each sample and experimental systematic errors.

The calculation results for stacked graphene-quartz absorbers are depicted in Fig. 4a and 4c. For simplicity, the graphene films are assumed to have the same parameters as per the initial calculation (Γ = 5 meV, *μ_c_* = 0.15 eV) which corresponds to a sheet resistance of 859 Ω/sq. The calculated reflection spectra in Fig. 4a have the same number of reflection zeros as the measured stacked units, which extends the absorption bandwidth but keeps the centre frequency around 148 GHz. A similar phenomenon exists in the absorption spectra in Fig. 4c, which show more absorption peaks and wider absorption bands as the layers increase. Mutual coupling of the Fabry-Perot resonators contributes to the multiple absorption peaks within the band. The measured results in Fig. 4b and 4d show a good agreement with the calculations, except for a small frequency shift of reflection zeros and an increased reflection within the band. The difference is possibly due to parameter errors and additional losses in the practical samples, as well as the small air-gap between adjacent units that induces multiple reflections. For the 5-unit stacked absorber, approximately 90% absorption can be achieved for 125–165 GHz, which indicates the practical millimetre wave absorber has a 28% fractional absorption bandwidth with the added benefit of optical transparency.

For a dielectric substrate, the resonance frequency will be decreased by increasing either the permittivity or thickness of the slabs. Moreover, the relative permittivity will affect the characteristic admittance of the dielectric according to equation (4). Thus, a decreased relative permittivity not only widens the bandwidth but also improves the absorption within the band (Supplementary Fig. S4). The most appropriate choice for achieving wide bandwidth and high absorption is a relative permittivity close to 1. However, in this paper, quartz was selected for fabrication simplicity and its high optical transparency. Certainly, the use of quartz substrates result in a reduced absorption performance compared to other low permittivity substrates.

There are two contributions to microwave absorption: dielectric and magnetic loss^41^, due to the imaginary parts of a material's complex permittivity and permeability. Pure graphene materials are found to contribute to microwave energy absorption mostly because of their dielectric loss rather than magnetic loss. Due to this imbalance of dielectric permittivity and magnetic permeability of graphene, impedance matching for microwave absorbing materials is difficult to achieve for pure graphene. As a result, magnetic materials such as Fe_3_O_4_ are added to graphene to result in high complex permeability values over the GHz range^41^. The performance of single-layer absorbers can be improved by increasing the permeability; however, known magnetic materials do not exceed several units^42^. Therefore broad-band non-magnetic absorbers employ multilayer absorbing structures or dielectrics with frequency dispersion of the permittivity.

In this work, in order to achieve optical transparency, we have not used multilayer graphene as a dielectric filler but as an ultra thin, lossy resistive component separated by the transparent quartz dielectric filler, backed by a ground plate, forming a Jaumann absorber. We aimed to match the impedance to free space by engineering the surface resistance characteristics of the multilayer graphene combined with the impedance transformation of the transparent dielectric filler. We are able to achieve absorption of 90% of the incident millimetre wave without necessarily taking permeability into consideration.

Although data on oblique incidence is not presented, an analytical prediction can be obtained which we have validated in the normal incidence case (Supplementary Fig. S5 and Fig. S6). We observe that incident angles *θ* < 30° do not have a significant impact on the absorption spectra. However, when *θ* > 60° absorption is reduced and the magnitude variations become significant for TE-polarized waves, whilst they improve for TM-polarized cases. We also observe that absorption bandwidth increases significantly when the relative permittivity is reduced from *ε_r_* = 3.8 to *ε_r_* = 1.1; suggesting that a dielectric with low relative permittivity is required to achieve both wide bandwidth and high absorption.

## Discussion

By stacking nanometre-thick multilayer graphene films and millimetre-thick quartz slabs, we have developed a facile and optically transparent broadband millimetre wave absorber. This absorber has been modelled using transmission line theory and an analytical expression has been derived to predict its performance.

Each graphene-quartz structure forms a Fabry-Perot resonator. When backed with a ground plate, absorption peaks or reflection zeros occur at odd numbers of the fundamental resonant frequency. We have determined that the sheet resistance of the graphene is an important factor for determining the input impedance and free space impedance matching; here the multilayer CVD graphene films were used to achieve the required resistivity through a repeated transfer process developed to minimise PMMA residues.

When multiple graphene-quartz samples are stacked together, the mutual coupling of the Fabry-Perot resonators introduces multiple absorption peaks in each band leading to broadband absorption. A 5-unit stacked graphene-quartz absorber seems adequate to counterbalance the absorption performance and the manufacturing complexity. In addition, the permittivity and thickness of the dielectric slabs provide additional degrees of freedom to adjust the bandwidth and in-band performance. Furthermore, for the case of oblique incidence, the absorber is found to be suitable for angles <30° for TE-polarized waves while good for all angles for TM-polarized waves. In order to achieve excellent absorption for wide incidence angles under TE-polarization, pyramidal or inhomogeneous structures may be used for future work.

The measurements of transparent graphene-quartz absorbers have been carried out using a millimetre wave reflectometer across the 110–170 GHz band. Our measured results validate that multilayer graphene achieves reduced sheet resistance assessed by tuning the chemical potential in the calculation, and 5-unit stacked absorber achieves 90% absorbance with a 28% fractional bandwidth. Moreover, the derived analytical formulae predicted the periodic absorption spectra over a wide frequency region, and the proposed absorber can be designed to meet different requirements with the added benefit of optical transparency.

## Methods

### CVD Graphene synthesis

Graphene was grown by thermal chemical vapour deposition in a cold-walled, commercially available reactor (Aixtron Black Magic). 500 nm-thick Cu was magnetron sputtered onto 200 nm thermally oxidised Si-100 four inch wafers which were subsequently annealed at 850°C for 30 min in a 20:1500 standard cubic centimetres per minute H_2_:Ar atmosphere at 4 mbar. Graphene growth was initiated by introducing 7 sccm CH_4_ (99.9%) under maintained H_2_ (99.98%) and Ar (99.998%) dilution. All samples were cooled to <300°C under ultra-high purity N_2_ (99.999%) before venting to atmospheric pressure. Temperatures were monitored using two type K bimetallic thermocouples and a surface infrared interferometer. Pressures and temperatures were accurate to within ±0.1 mbar and ±1°C, respectively.

### Transfer of monolayer and stacked multilayer graphene

The graphene on Cu/SiO_2_/Si samples were first diced to 17 mm × 8.5 mm and then cast with 200 nm poly (methyl methacrylate)-(PMMA 950 A4) and were annealed at 180°C for 1 minute. Substrates were then immersed in an aqueous ammonium persulfate ((NH_4_)_2_S_2_O_8_, 2.2 g/100 mL DI) for 15 hours to etch the Cu catalyst. The floating PMMA-graphene films were then transferred into DI water baths using microscope glass slides and rinsed several times to remove the etchant. For monolayer graphene, the PMMA-graphene film was transferred to Saint-Gobain Spectrosil® 2200 Optical Fused Quartz substrates, and dried at atmospheric pressure for 24 hours. For monolayer samples the films were transferred to occupy only half side of the quartz substrate, leaving the other half exposed for UV-VIS spectro-photometry baseline calibration. For multilayer samples the films were transferred onto slightly larger diced wafer substrate pieces, in order to make PMMA/bilayer graphene stacks. The bilayer stacked substrates were etched again in (NH_4_)_2_S_2_O_8_ and were rinsed and transferred onto diced wafer quartz substrates, followed by acetone washes to remove the PMMA. The remaining PMMA/bilayer graphene films were transferred onto larger substrates and the process was repeated until stacked samples of up to 5 layers were processed.

### Millimetre wave reflectometer measurement

A HP N5244A vector network analyser and its millimetre wave extension heads were used to drive the reflectometer (Fig. 2a). The vertically polarized incident waves were emitted from the feed horn then filtered by a horizontal grating and reflected by an ellipsoidal reflector. A 45-degree grating was utilized to convert the vertically polarized wave to a horizontally polarized one by rotating it twice. The horizontal grating then reflected the wave to the feed horn of the horizontal receiver. Both the transmitter and receiver were connected to the vector network analyzer which calculated the reflection coefficient between the reflected wave and the incident wave.

## Author Contributions

Y.H. proposed and supervised the project. W.I.M. advised on the project. B.W. carried out the absorber design and data analysis, M.T.C. synthesised the CVD graphene. H.M.T. fabricated the multilayer graphene samples and carried out sheet resistance and UV-Vis optical transmission measurements. B.Y. set up the millimetre wave measurement system. B.W., B.Y. and M.N. performed the measurements. Finally, the manuscript was prepared by B.W. and H.M.T. and revised by Y.H., M.T.C. and W.I.M.

## Supplementary Material

Supplementary Information Supplementary
