# Supplementary material for: Experimental demonstration of a transparent graphene millimetre wave absorber with 28% fractional bandwidth at 140 GHz
Source: Sci Rep. 2014 Feb 19;4:4130. doi: 10.1038/srep04130 (PMC3928574; doi:10.1038/srep04130)
Supplement: Supplementary Information — Supplementary [file srep04130-s1.doc]

**Supplementary Information**

**Experimental demonstration of a transparent graphene millimeter wave absorber with 28% fractional bandwidth at 140 GHz**

Bian Wu^1,3^, Hatice M. Tuncer^2^, Majid Naeem^1^, Bin Yang^1,4^, Matthew T. Cole^2^, William I. Milne^2^,Yang Hao^1,*^

*^1^ School of Electronic Engineering and Computer Science, Queen Mary University of London, London, E1 4NS, United Kingdom;*

*^2^ Department of Engineering, University of Cambridge, 9 JJ Thomson Avenue, Cambridge, CB3 0FA, United Kingdom;*

*^3^ School of Electronic Engineering, Xidian University, Xi’an, 710071, China;*

*^4^ Engineering, Sports & Sciences Academic Group, University of Bolton, Deane Road, Bolton, BL3 5AB, United Kingdom.*

*^*^Correspondence should be addressed to Y.H. (email: y.hao@qmul.ac.uk).*

**The Supplementary Information includes:**

**Supplementary Figures S1 – S6.**

| **a** | 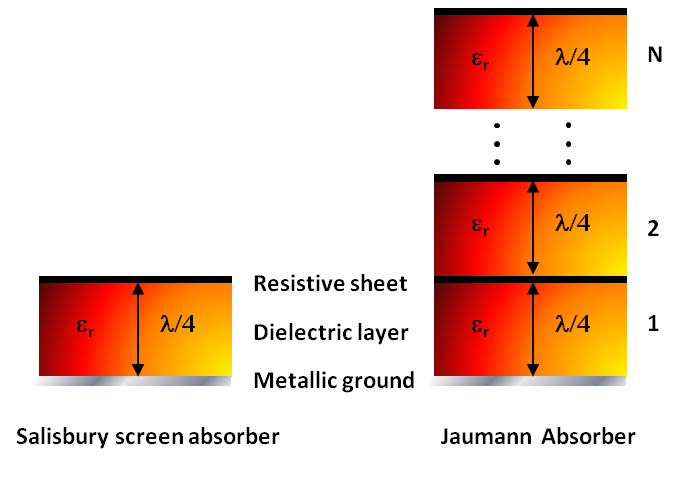 |
| --- | --- |
| **b** |  |

**Figure S1 | Schematic diagram of typical resonant absorbers and the reflection spectra of a single graphene-dielectric absorber.** (**a**) Salisbury screen absorber and Jaumann absorber, equivalent to a multilayer Salisbury screen. (**b**) Reflection spectra of a single graphene-dielectric Salisbury screen absorber, which shows periodic reflection zeros (absorption peaks) at zero-phase points, occurring at f_i_ = (2i-1)f_0_. The scattering rate and chemical potential of graphene are set to Γ = 5 meV and μ_c_ = 0.3 eV at T = 300 K. The relative permittivity of the 1.3 mm thick dielectric slab is ε_r_ = 3.8.

| **a** |  |
| --- | --- |
| **b** |  |

**Figure S2 | Reflection phases and absorption spectra of N = 1-3 unit stacked graphene-dielectric absorbers.** (**a**) Reflection phase spectra show that the absorption peaks occur at the zero crossing points as the phase changes from positive to negative angles. (**b**) Absorption spectra showing the peak locations. The scattering rate and chemical potential of graphene are set to Γ = 5 meV and μ_c_ = 0.2 eV at T = 300 K; relative permittivity and thickness of the dielectric slabs are ε_r_ = 1.1 and h = 1 mm.

| **a** |  |
| --- | --- |
| **b** |  |

**Figure S3 | Calculated absorption spectra of the 3-unit absorber and its electric-field distribution at absorption peaks (modes).** (**a**) Absorption spectra with varying chemical potentials of the graphene sheets. There are three absorption peaks in the first band, labelled as mode A, B, C, which are induced by the mutual coupling of the three Fabry-Perot resonators (Γ = 5 meV, T = 300 K, ε_r_ = 1.1, h = 1 mm). (**b**) Electric-field distribution when μ_c_ = 0.2 eV; mode C has the shortest wavelength corresponding to the highest resonant frequency. The E-field magnitude inside the absorber has diminished due to high absorption.

| **a** |  |
| --- | --- |
| **b** |  |

**Figure S4 | Influence of dielectric parameter variations on absorption spectra of the 3-unit graphene-dielectric absorber.** (**a**) Impact on bandwidth (BW) and magnitude of absorption is shown for various relative permittivity values for different materials; foam (ε_r_ = 1.1), PMMA (ε_r_ = 2.7) and quartz (ε_r_ = 3.8) with the dielectric thickness set to h = 1 mm. (**b**) Thickness variation at a fixed relative permittivity (ε_r_ = 1.1) has an impact on bandwidth only.

| **a** |  |
| --- | --- |
| **b** |  |

**Figure S5 | Absorption spectra of the 5-unit absorber at oblique incidence angles (ε_r_ = 1.1).** (**a**) TE-polarization. (**b**) TM-polarization. The bandwidth increases with increasing incidence angles for both polarizations. The absorption is reduced significantly and the absorption magnitude variation is more prominent for θ > 60⁰ with TE-polarization.

| **a** |  |
| --- | --- |
| **b** |  |

**Figure S6 | Absorption spectra of the 5-unit absorber at oblique incidence angles (ε_r_ = 3.8).** (**a**) TE-polarization. (**b**) TM-polarization. The bandwidth increases, the absorption is reduced and the absorption ripple variation is insignificant for θ > 60⁰ TE-polarization. For TM-polarization, absorption increases and the amplitude of ripples decrease for increasing angle of incidence.
